# Impaired tongue motor control after temporomandibular disorder: A proof‐of‐concept case‐control study of tongue print

**DOI:** 10.1002/cre2.549

**Published:** 2022-02-27

**Authors:** Caroline Alvarado, Audrey Arminjon, Clovis Damieux‐Verdeaux, Claire Lhotte, Chloé Condemine, Anne‐Sabine Cousin, Nicolas Sigaux, Pierre Bouletreau, Sébastien Mateo

**Affiliations:** ^1^ Cabinet de kinésithérapie Saint‐Alexandre Lyon France; ^2^ Hospices Civils de Lyon, Groupement Hospitalier sud Chirurgie maxillo‐faciale Pierre‐Bénite Cedex France; ^3^ Lyon Neuroscience Research Center, Trajectoires Team, CNRS UMR5292, INSERM U1028 Université de Lyon, Université Lyon 1 Lyon France; ^4^ Hospices Civils de Lyon Hôpital Henry Gabrielle, Plate‐forme Mouvement et Handicap Lyon France

**Keywords:** clinical assessment, mental representation, physiotherapy, rehabilitation

## Abstract

**Background:**

Temporomandibular disorder (TMD) perturbs the tongue motor control and consequently impairs oral function, but strength training reduces this impairment. However, tongue motor control is widely reduced to a matter of strength.

**Objectives:**

To investigate the accuracy of the tongue placement as a measure of tongue motor control in patients with TMD compared with age‐ and sex‐matched healthy participants.

**Material and Methods:**

This proof‐of‐concept case‐control study was prospective, observational, and part of the TMIQ study (NCT04102306). After pointing against a wood stick while maintaining the tongue as sharp as possible, the examinator drew the contour of the tongue print on the wood stick, which was then scanned for image analyses to compute the area for each participant using ImageJ.

**Results:**

A total of 94 participants were included, all patients with TMD (*n* = 47) diagnosed with myalgia, 61% with intra‐articular joint disorder accordingly to the DC/TMD. The median (IQR) tongue print area was 117 (111) mm^2^ for the TMD group and 93.5 (76.2) mm^2^ for the control group (*V* = 352, *p* = .04) and the median [95% confidence interval] difference was 25.4 [1.3; 51.0] mm². Overlapping of the 95% confidence intervals of the area evidenced no significant difference between the categories of the DC/TMD. The corrected each area–total correlation (*r* = .24) suggests a reasonably homogenous thus valid measure.

**Conclusion:**

The results suggest that TMD impairs the motor control of the tongue. Therefore, the sharpest tongue pointing test may constitute a simple and accessible clinical tool to assess the accuracy of tongue placement in TMD patients. The study was registered on ClinicalTrial.gov with identification number NCT04102306.

## 1 INTRODUCTION

The tongue plays an essential role during respiration (Cheng et al., 2008) oral transport, swallowing, and speech production (Hiiemae & Palmer, 2003). To achieve these roles, the tongue musculature allows myriad changes in the tongue shape enabled by arrangements of tongue muscle fibers in multiple axes, involving extrinsic and intrinsic muscles (Gaige et al., 2007; Takemoto, 2001). The movements of the tongue are essentially intrabuccal, thus not directly seen, and protocols have been established to screen tongue dysfunction (Gill & Fougeront, 2015) but remain subjective in nature (Youmans & Stierwalt, 1971). Tongue strength has been widely considered as an objective assessment of tongue function, including swallowing in healthy or elderly persons (Adams et al., 2014, 2015). A large body of evidence is available and has related tongue strength impairments and dysfunctions in both masticatory and swallowing in patients with the chronic temporomandibular disease (TMD) (Ferreira et al., 2014; Marim et al., 2019; Rosa et al., 2020; Wright, 2009). A recent study has shown that asymptomatic people (i.e., with no TMD) significantly improved their tongue strength after three sessions of action observation training before physically practiced exercises of tongue strengthening, unlike asymptomatic people who practiced physical exercises alone or in combination with motor imagery before the exercises. In their study, the authors used a tongue depressor as a means to induce resistance against lateral, upward, and forward movements of the tongue. Albeit several validated tools exist for assessing tongue strength (Yoshikawa et al., 2011), we believe that the tongue depressor could be used as a tool to assess the hypothesis—based on the clinical experience acquired during TMD rehabilitation—that the motor control of the tongue would be significantly decreased in patients with TMD during the completion of the sharpest tongue pointing test compared with non‐TMD participants. To test this hypothesis, the present study investigated whether the tongue print area against the tongue depressor was statistically significantly larger in patients with TMD compared with age‐ and sex‐matched healthy participants (i.e., with no history of TMD).

## 2 METHODS

### 2.1 Study design

This case‐control study was prospective and observational and conform to the Declaration of Helsinki and to the STROBE guidelines (von Elm et al., 2007). Approval was obtained from the regional ethics committee (CPP Sud‐Ouest et Outre‐Mer III 2018‐A02195‐50). Print tongue measurement was performed immediately after inclusion in the “Assessing Motor Imagery Ability of Tongue and Mouth in Subjects With and With no TMD” TMIQ study, aiming to assess the motor imagery ability of tongue and mouth in subjects with and without TMD (https://clinicaltrials.gov/NCT04102306) (Alvarado et al., 2022).

### 2.2 Setting

All patients with TMD were recruited from the physiotherapy facility (private practice) *Cabinet Saint Alexandre* (Lyon, France) that exclusively receives patients for TMD rehabilitation. Control participants were recruited within the family environment of either the patients with TMD or the authors or from the authors' professional entourage. To address our objectives, the present study was restricted to tongue prints only.

### 2.3 Participants

In the TMIQ study, French‐speaking volunteers aged between 18 and 75 years were considered for inclusion. To be included in the TMD group, (i) a physician independent of the study diagnosed TMD and delivered a written prescription for TMD rehabilitation before the study enrollment, (ii) and the physiotherapist classified the type of pain and intra‐articular TMD during screening according to the diagnostic criteria for the temporomandibular disorders (DC/TMD); imaging was not used (Schiffman et al., 2014). Healthy subjects included in the control group were age‐ and sex‐matched to TMD participants. The DC/TMD was only completed for patients with TMD. Exclusion criteria were the presence of a short lingual frenulum (Marchesan, 2005), lingual immaturity (Jouannaud et al., 1972), and/or peripheral facial palsy, a history of orthognathic surgery or facial fracture during the 6 previous months, the current participation in another study to prevent any experimental bias; patients with TMD were not included in the control group.

### 2.4 Variables

The print tongue was the primary outcome considered in this study. Since pain may have a non‐negligible effect on the measure, the intensity of the pain was recorded by interviewing the participant using a visual analogic scale ranging from 0 (*no pain*) to 10 (*maximal imaginable pain experienced*). In addition, maximal mouth opening was measured once using a caliper as recommended for both control participants and patients with TMD (Best et al., 2013); this measure was subsequently used for DC/TMD classification of intra‐articular TMD for patients.

### 2.5 Measurement

**Figure 1:**
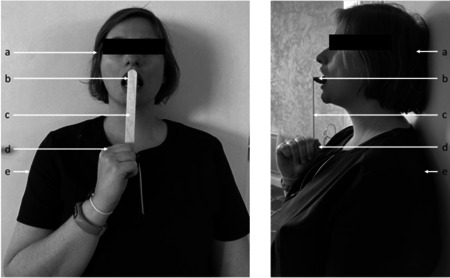
Installation and instructions for the sharpest tongue pointing test. a: Head against the wall; b: proximal extremity of the stick held vertically in front of the participant's mouth. Participants received the following instruction: pushing for 2–3 s the wood tick against their tongue while being instructed by the examiner to maintain the tongue as sharp as possible (i.e., resisting with the sharpest tongue to the slight pressure applied by the wood stick); c: wood stick held vertically; d: hand against the trunk holding firmly and vertically the wood stick; e: trunk against the wall

The sharpest tongue pointing test was performed immediately after inclusion in the TMIQ study. The measurement was achieved before the administration of the motor imagery questionnaires. Participants sat and maintained their back and head against a wall to prevent any trunk or head forward movements that could be performed during the measurement. Participants were instructed to open their mouth sufficiently to point with the tongue with no contact between the tongue and the teeth. Participants held an 18‐mm‐large wood stick vertically and firmly the distal extremity of the stick with one hand placed against the trunk so that the proximal extremity of the stick was placed in front of their mouth; pushed for 2–3 s, the wood tick against their tongue while being instructed by the examiner to maintain the tongue as sharp as possible (i.e., resisting with the sharpest tongue to the slight pressure applied by the wood stick) with no long teeth contact. Immediately after, the examiner drew the contour of the tongue print on the wood stick with a pen before the drying of the saliva. In case the tongue print exceeded the wood stick width, the measure was repeated while holding the stick horizontally. In case of forward movement of the trunk or head (i.e., contact lost between the head or trunk and the wall) or contact between the tongue and the long teeth, the measure was repeated (Figure 1). Finally, the wood stick with the tongue print was scanned for image analyses, and computing of the tongue print area of each participant was performed using the opensource software ImageJ 1.52k (Wayne Rasband, National Institutes of Health, http://imagej.nih.gov.ij). The area of the tongue print was modeled using the function “analyze particles” that computed the ellipse area along with major and minor axes of the ellipse using an overlay mask. For graphic representation, the tongue print background of each participant (i.e., the wood stick) was removed using the GNU Image Manipulation Program Gimp 2.8.22 (https://www.gimp.org/). Finally, tongue prints were sorted by size from the smallest to the largest area for each group independently. In addition, the program Scientific Python Development Environment Spyder 3.3.1 (https://www.spyder-ide.org) was used to draw three contours corresponding to the median area of the tongue print, the lower and the upper limit of the 95% confidence interval (CI) of the median, knowing the median and the 95% CI of the major and minor axes of the ellipse for each group. We used the pain measurements from the TMIQ study that were measured during the administration of the questionnaire. Briefly, the participant was instructed to imagine a total of five imagined movements of the mandible (*n* = 3) and of the tongue (*n* = 2). The presence of pain was verified by the use of the visual analogic scale. We used the median score of the five movements for each participant to investigate the possible influence of pain on the tongue print area.

### 2.6 Bias

Participants received no specific information and therefore had no knowledge of the hypothesis of this study but, because the tongue print measurement required active movements from the participant and supervision from the physiotherapist examiner, neither the participant nor the assessor was blinded to the evaluation; therefore, to limit a possible assessment bias each physiotherapist assessed exclusively participants within the same group (i.e., patients with TMD were exclusively assessed by C. A., A. A., C. L., C. C., and control participants by C. D.‐V. and S. M.). All physiotherapists received specific training and have more than 5 years of experience in TMD rehabilitation (which includes tongue training).

### 2.7 Study size

The sample size corresponded to another computation from the TMIQ study, for which a total of 94 participants was needed to be included (i.e., 47 participants per group) (Alvarado et al., 2022).

### 2.8 Statistical methods

Shapiro test revealed that data were normally distributed only regarding the maximal mouth opening but not regarding the participant age and the tongue print area. Therefore, maximal mouth opening was expressed as mean (95% CIs); age and tongue print area as median (interquartile range, IQR). Then, the tongue print area that exceeded the median area plus 3× median absolute deviation of the corresponding group was considered as an outlier (Leys et al., 2013) and subsequently removed from the analysis and visual representations. Groups were compared regarding the participant age and the tongue print area using the paired Wilcoxon's test; maximal mouth opening using *t* test. Statistical significance was set at 5% (*p* < .05). In case the Wilcoxon test showed a significant difference between groups for the primary outcome, the median difference between groups and its 95% CI was computed (Bauer, 1972). Furthermore, the effect size of the Wilcoxon test was computed and reported as *r* value and its 95% CI (Tomczak & Tomczak, 2014). Finally, since power ≥80% is considered as sufficient to ensure an adequate sample size (Cohen, 1992), the power of the statistical comparison between groups was searched for the tongue print area; this required to (i) verify that the tongue print area corresponded to a gamma distribution (Rigby et al., 2019), (ii) compute the shape and the scale parameters for each group (Anderson & Ray, 1975), (iii) repeat 10,000 times with simulated gamma distribution knowing the shape and the scale parameters but modulating the number of participants until obtaining a percentage of detected significant difference between group ≥80% (Hochster, 2008). In addition, the validity of the sharpest tongue pointing test was searched separately for patients with TMD and control participants using the alpha function of the psych package to compute the *corrected each area–total correlation* (Field et al., 2012); *r* values between 0.2 and 0.4 suggest reasonably homogenous measure thus can be considered as valid (Piedmont, 2014). Finally, exploratory analyses were conducted computing for each category of the DC/TMD the median and 95% CI (calculated from the median) for the primary outcome; overlapping tongue print area 95% CI indicated no significant difference between categories of the DC/TMD therefore suggesting no influence of the type of pain or the type of intra‐articular disorder on tongue motor control. We also investigated whether the ability to open the mouth was related to the tongue motor control by computing separately for the control and TMD groups' Pearson's correlation between maximal mouth opening and tongue print area. All statistical analyses were performed using R 4.0.0.

## 3 RESULTS

**Figure 2:**
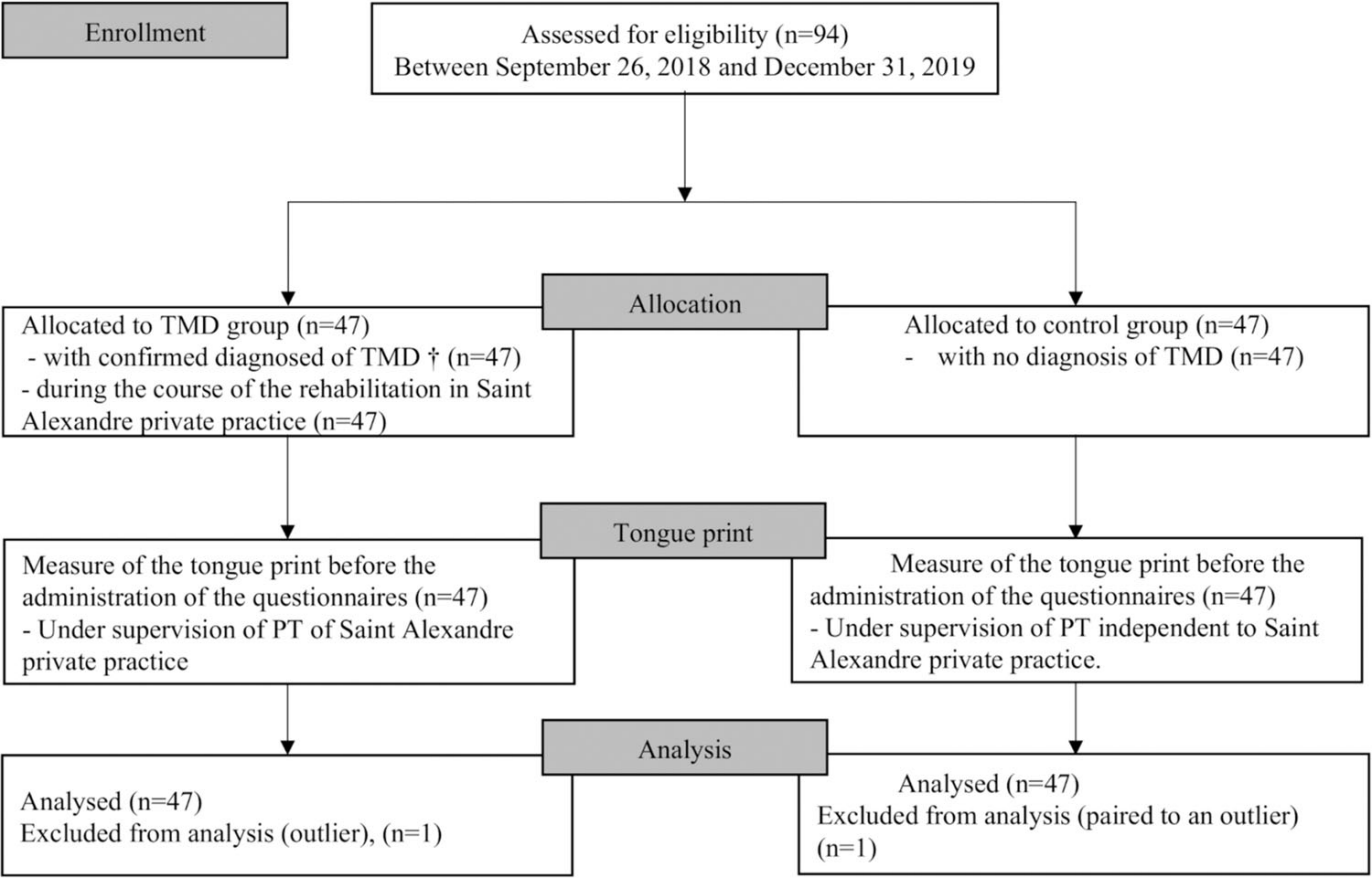
Flowchart summarizing the different steps of the study, including enrollment, allocation, measurement, and analysis. ^†^Confirmed medical TMD diagnostic before the study inclusion and completion of the DC/TMD classification by the physiotherapist at Saint Alexandre private

All included participants completed the task; inclusions started on September 26, 2018, and the study was completed on December 31, 2019 (see Figure 2).

### 3.1 Participant characteristics

There were 47 participants, among whom 32 (68%) were female, in each group. Accordingly to the DC/TMD, all patients reported myalgia—local myalgia (*n* = 6, 13%), myofascial pain (*n* = 21, 45%), myofascial pain with a referral (*n* = 20, 23%); among the 47 patients with TMD, 17 (36%) had no intra‐articular disorder, 18 (38%) had a disk displacement with reduction, 10 (21%) had a disk displacement without reduction and with a limited opening, and 2 (4%) a degenerative joint disease (Table 1). The median (IQR) age of the patients with TMD was 34 (21) years and was not significantly different from the one of the age‐matched healthy participant group [35 (24); *V* = 512, *p* = .9]. The mean and 95% CI maximal mouth opening of the patients with TMD was 44 [41; 46] mm not significantly different to the one of control participants 44 [42; 45] mm, *t*_(78)_ = 0.1, *p* = .9. Therefore, all participants, especially in the TMD group, were able to open their mouth wide enough to point with the tongue with no contact with the teeth and no associated pain, which was confirmed by no reported pain during the measurement (see also Table 1).

**Table 1:** Participant's characteristics and tongue print area accordingly to the DC/TMD

	Myalgia			Intra‐articular joint disorders			
	Local	Myofacial^a^	Myofacial^b^	noDD	DDwR	DDwoRwLO	DJD
Number (%)	6 (13)	21 (45)	20 (43)	17 (36)	18 (38)	10 (21)	2 (4)
Gender (M/F)	3/3	13/8	16/4	11/6	10/8	9/1	2/0
Age (years)	28 [20; 65]	28 [23; 42]	40 [31; 52]	36 [31; 53]	28 [25; 41]	30 [22; 52]	39 (5)^c^
MMO (mm)	45 [36; 50]	44 [39; 51]	45 [39; 46]	45 [40; 47]	50 [45; 54]	36 [29; 39]	32 (10)^c^
Area (mm^2^)	143 [55; 212]	113 [81; 182]	119 [74; 211]	149 [127; 212]	98 [74; 133]	81 [51; 189]	152 (76)^c^

*Note*: All values are expressed as median and [95% confidence interval] otherwise explicitly stated. All confidence intervals showed overlapping evidencing no significant differences between myalgia categories and intra‐articular joint disorders for none of the considered parameters (age, MMO, area).

Abbreviations: 95% CI, 95% confidence interval; DDwoRwLO, disk displacement without reduction with limited opening; DDwR, disk displacement with reduction; DJD, degenerative disk disorder; F, female; M, male; MMO, maximal mouth opening; noDD, no disk displacement; Number, number of patients among 47.

^a^ Myofacial pain.

^b^ Myofacial pain with referral.

^c^ Interquartile range was computed instead of 95% CI since data of two participants was insufficient using the MedianCI function of the DescTools Package of R.

### 3.2 Tongue print area

**Figure 3:**
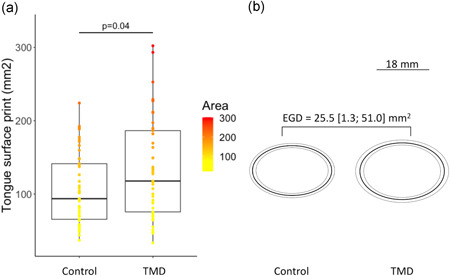
Significantly larger tongue print area for the temporomandibular disorder (TMD) group compared with the control group. (a) Boxplot representing the area of the tongue print where bottom, top, and middle lines in the box represent 1st, 3rd quartiles, and median, respectively, and the two vertical bars extend to the minimum to maximum values (b); ellipses representing the median (dark line) and the lower and upper limit of the 95% confidence interval of the median (light lines and gray area) of participants of the control group (left panel) and TMD group (right panel). The estimated median group difference (EGD) and its 95% confidence interval was a tongue print area larger by 25.5 [1.3; 51.0] mm^2^ for patients with TMD as compared with control participants

**Figure 4:**
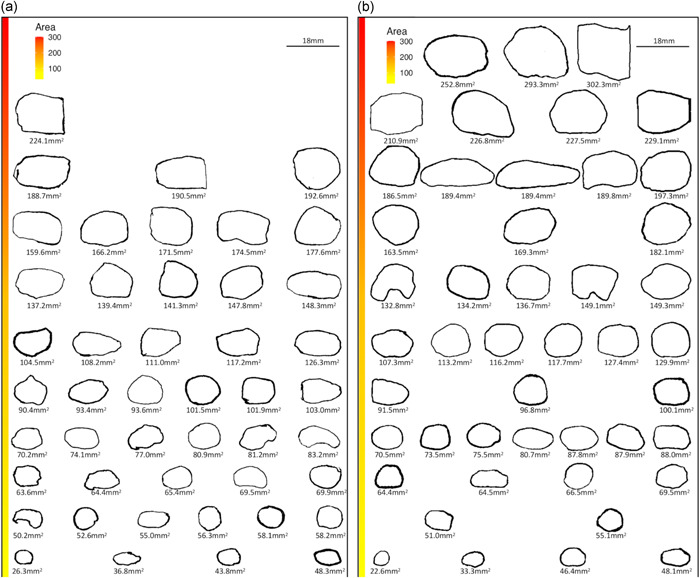
Tongue print sorted by size for each participant of the (a) control group and (b) temporomandibular disorder (TMD) group

One TMD participant was considered as an outlier (429.9 mm^2^) and was therefore removed from the analysis and visual representations, as was the matched control participant. The median (IQR) tongue print area was 117 (111) mm^2^ for the TMD group and 93.5 (76.2) mm^2^ for the control group, the tongue print area of the TMD group was significantly larger (*V* = 352, *p* = .04) and the median [95% CI] difference was 25.4 [1.3; 51.0] mm^2^. This significant difference represented a moderate effect size with an *r* value [95% CI] of 0.30 [0.04; 0.54] (Figures 3 and 4). For the 46 participants analyzed in each group, the Wilcoxon test was underpowered with a 48%. To achieve an adequate power of 80%, 100 participants per group should be included. The *r* value of the *corrected each area–total correlation* was equal to 0.24, suggesting a reasonably homogenous measure thus can be considered as valid. Furthermore, all 95% CI of the tongue print area overlapped, indicating no significant difference, thus suggesting no effect of the DC/TMD category on tongue motor control (Table 1). There was also no significant correlation between maximal mouth opening and tongue print area suggesting that tongue motor control was independent of the capacity of maximal mouth opening for either control participants or patients with TMD (see Figure S1).

## 4 DISCUSSION

The present study showed that patients with TMD had a significantly larger tongue print area against a tongue depressor compared with healthy participants, suggesting that TMD impairs the motor control of the tongue. Importantly, because no pain was reported while performing the sharpest tongue pointing test, it is very unlikely that pain had an influence on this relationship. This is consistent with other studies that have shown a relationship between tongue strength deficits and function impairments, including swallowing, speech production, and intelligibility (McLoon & Andrade, 2013). However, one can notice that the tongue is much less susceptible to develop strength compared with appendicular muscle, as demonstrated in studies that have shown that the rat tongue motor units produced 100–1000‐fold less force than the rat appendicular motor units (McLoon & Andrade, 2013), and that supra‐normal subjects who had acquired a high skill level with their tongue (e.g., trumpet players and high school debaters who were able to speak intelligibly at rates much faster than normal) displayed no difference in strength compared with normal subjects (Robin et al., 1992). Thus, the tongue strength may not be the most relevant outcome to fully assess the tongue function (including accuracy). Indeed the results from the sharpest tongue pointing test performed herein suggested that a measure of the tongue motor control could complete the already validated strength measurement tools (e.g., Intra Iowa Oral Performance Instrument, IOPI) (Adams et al., 2014), as asking to point against the tongue depressor with an as‐sharp‐as‐possible tongue constitutes a simple and accessible clinical tool to assess the accuracy of the tongue placement. This may further reflect the requirement of accurate placement of the tongue during its different functions with no visual feedback, and as such constitutes an accurate measurement of the tongue motor control. This relatively simple and objective measurement could also complete the clinical protocols used to investigate the tongue function (Gil & Fougeront, 2015), or the ultrasound measures to analyze tongue motion during its function (Stone, 2005), and be used as a tool to monitor the effect of rehabilitation (La Touche et al., 2020).

Nevertheless, this study has several limitations. First, albeit the significant difference found between groups regarding the tongue print area, the study was underpowered essentially because the measure was ancillary, that is conducted during another study (TMIQ) for which the sample size was computed. This result should be considered as preliminary and the difference reported representing a moderate effect size must be confirmed by future studies with adequate statistical power (i.e., including at least 100 participants per group). Furthermore, albeit results suggest a valid measure, the psychometric properties of the tongue print measurement, including the reliability of the measurement, could not be determined because of the study design (i.e., one single tongue print assessment).

Future studies should confirm the validity of this measure and investigate its psychometric properties related to this measurement, including the control of the tongue pressure during the measurement, corroborate the absence of effect of the different categories of the DC/TMD, and consider the sharpest tongue pointing test in conjunction with other validated tools, namely, but non‐exhaustively, the IOPI (Adams et al., 2013) and/or the ultrasound tongue measurements (Stone, 2005). Moreover, future studies should consider documenting the participant personality and motivation to verify the absence of difference in these parameters in addition to the absence of difference in age and sex between groups to control a possible Hawthrone effect and prevent an assessment bias. In addition, to confirm the clinical interest of the tongue area measurement, future studies could investigate the kinetics of change of the tongue area measurement (i.e., tongue motor control) in response to TMD rehabilitation as a function of the severity and DC/TMD category of the TMD and consider investigating changes in impairment of functions during which the tongue is essential. Finally, neuroimaging investigations could be of interest to explore the brain correlates of these behavioral modifications (Corfield et al., 1999; Leonard et al., 2017).

## 5 CONCLUSION

The results suggest that TMD impairs the motor control of the tongue. Therefore, asking to point against the tongue depressor with an as‐sharp‐as‐possible tongue constitutes a simple and accessible clinical tool to assess the accuracy of the tongue placement in TMD patients.

## CONFLICTS OF INTEREST

The authors declare no conflicts of interest.

## AUTHOR CONTRIBUTIONS

All authors participated in the study conceptualization, data curation, writing of the original draft, and revision of the manuscript. Both Sébastien Mateo and Caroline Alvarado supervised the study. Sébastien Mateo performed the analyses. All authors approved the final version before submission.

## Supporting information

Supporting information.

Supporting information.

Supporting information.

## Data Availability

Data used for analysis has been submitted as supportive information and is available as an additional file.
