# Supplementary material for: Impaired tongue motor control after temporomandibular disorder: A proof‐of‐concept case‐control study of tongue print
Source: Clin Exp Dent Res. 2022 Feb 27;8(2):529–36. doi: 10.1002/cre2.549 (PMC9033548; doi:10.1002/cre2.549)
Supplement: Supplementary file 3 — Supporting information. [file CRE2-8-529-s002.docx]

**Supplementary Figure 1.** No significant correlation between the maximum mouth opening and the tongue print area for either patients with TMD (A) or control participants (B). Data for 47 patients with TMD and 40 control participants (i.e., 5 missing data for maximal mouth opening) are presented.
